# Dynamically induced robust phonon transport and chiral cooling in an optomechanical system

**DOI:** 10.1038/s41467-017-00247-7

**Published:** 2017-08-07

**Authors:** Seunghwi Kim, Xunnong Xu, Jacob M. Taylor, Gaurav Bahl

**Affiliations:** 1 Mechanical Science and Engineering, University of Illinois at Urbana-Champaign, Urbana, Illinois 61801 USA; 2 Joint Quantum Institute, University of Maryland, College Park, Maryland 20742 USA; 3 Joint Center for Quantum Information and Computer Science, National Institute of Standards and Technology, Gaithersburg, Maryland 20899 USA

## Abstract

The transport of sound and heat, in the form of phonons, can be limited by disorder-induced scattering. In electronic and optical settings the introduction of chiral transport, in which carrier propagation exhibits parity asymmetry, can remove elastic backscattering and provides robustness against disorder. However, suppression of disorder-induced scattering has never been demonstrated in non-topological phononic systems. Here we experimentally demonstrate a path for achieving robust phonon transport in the presence of material disorder, by explicitly inducing chirality through parity-selective optomechanical coupling. We show that asymmetric optical pumping of a symmetric resonator enables a dramatic chiral cooling of clockwise and counterclockwise phonons, while simultaneously suppressing the hidden action of disorder. Surprisingly, this passive mechanism is also accompanied by a chiral reduction in heat load leading to optical cooling of the mechanics without added damping, an effect that has no optical analog. This technique can potentially improve upon the fundamental thermal limits of resonant mechanical sensors, which cannot be attained through sideband cooling.

## Abstract (web summary)

Chiral transport can provide robustness against disorder, resulting in improved resonant modes for sensing and metrology. Here, Kim et al. demonstrate chiral phonon transport, disorder suppression and anomalous cooling without damping in an asymmetrically-pumped optomechanical system.

## Introduction

Efforts to harness the optical and mechanical properties of achiral resonators are leading to new approaches for quantum noise limited sources^1–3^, preparation of quantum states of matter^4–7^, and ultra-high precision metrology^8–11^. Since all these efforts are aided by long coherence times for resonant excitations, they are fundamentally limited by structural disorder, even in systems with high symmetry, and by thermal noise in the mechanics. While optomechanical sideband cooling can lower the effective temperature of a mechanical oscillator, it does not modify the heat load, and thus does not fundamentally modify the contribution of thermal noise for, e.g., sensing or transduction^10, 11^. Surprisingly, the chiral edge states of topological insulators—in which different parity excitations travel in different directions—can provide improved transport properties, giving rise to unique physics ranging from nonreciprocal wave propagation to disorder-free transport in quantum Hall systems^12–16^. At the same time, inducing nonreciprocal behavior by breaking parity symmetry in achiral non-topological devices forms the basis for circulators^17, 18^ and recent proposals for optomechanical isolation^14, 16, 19, 20^. However, experiments to date on nonreciprocal optomechanical devices^19–21^ have focused entirely on optical behavior, and there has been no direct exploration on the chiral nature of propagating phonons in such systems, nor on the disorder tolerance induced through chirality.

Here we show how to optically impart chirality to achiral mechanical systems. Our approach results in disorder-less transport of sound, simultaneously improving the isolation of phonon modes from their bath and lowering their heat load without added damping^22^. We use a particularly simple class of systems for examining chiral behavior: passive devices with degenerate forward- and backward-propagating modes, such as ring cavities and whispering-gallery resonators (WGRs). The modes of these structures can alternatively be described as opposite parity pairs having clockwise (+ or cw) and counterclockwise (− or ccw) pseudo-spin of circulation^14^. Chiral here indicates pseudo-spin-dependent behavior in the system. Disorder breaks parity conservation in WGRs, preventing chiral behavior and leading to the additional loss of energy in high-*Q* modes via pseudo-spin flips and scattering into bulk modes, both optically^23^ and mechanically^24, 25^. Recent work has shown that asymmetric optical pumping of one pseudo-spin direction in WGRs explicitly introduces chiral behavior for photons with the assistance of even weak optomechanical coupling^19^. In this work, we demonstrate that this induced symmetry breaking in fact imparts parity-dependent behavior throughout the system, the chiral echoes of which are observable across its mechanical properties as the system develops robustness to parity-breaking disorder.

We observe three significant phononic chiral effects. First, cw and ccw phonons experience dramatic chiral optomechanical cooling. While this may be anticipated from past experiments on Brillouin optomechanical coupling with traveling phonons^19, 26^, such chiral phonon propagation has never been experimentally reported. Second, the optomechanical damping selectively provided to cw phonons results in mitigation of the disorder-induced loss for phonons having the opposite (ccw) pseudo-spin—effectively a phononic analog of the quantum Zeno effect. Finally, while the cw phonon modes experience conventional optomechanical cooling^4–6, 26–28^, an isolated high-*Q* ccw mode simultaneously experiences a reduction in damping and a reduction in temperature. This result reveals a surprising form of optomechanical cooling that occurs through chiral refrigeration of the thermal bath composed of the (cw) bulk mechanical modes of the system.

## Results

### Modal relationships in a whispering-gallery resonator

**Fig. 1:**
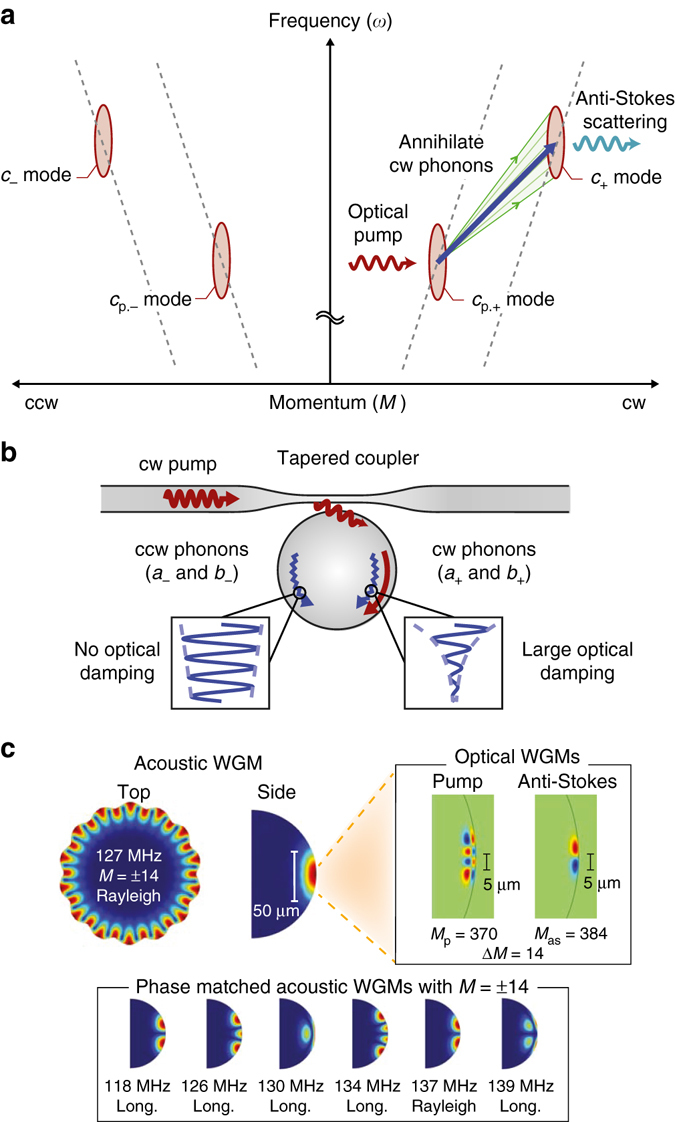
Chiral cooling and modal relationships in a whispering-gallery resonator. **a** Configuration of the two requisite optical whispering-gallery modes (WGMs) with cw (+) and ccw (−) degeneracy is illustrated in (*ω*, *M*) space. Integer *M* describes the azimuthal momentum of WGMs. Anti-Stokes Brillouin scattering from cw pumping of the lower mode annihilates only cw phonons of all phase-matched dispersion relations, while ccw phonons remain nominally unaffected, thus breaking parity symmetry in the system. The optical modes are illustrated elongated in *ω*-space due to the extremely large phase velocity of photons in comparison to phonons. **b** Directional optical interface to the resonator modes is achieved via tapered optical fiber. Unidirectional optical pumping results in dramatic chiral damping of the phonons. *a*_±_ and *b*_±_ phonon modes are described in Fig. 3. **c** Our experiment is performed using a high-*Q M* = ±14 acoustic WGM (*a*_±_) using two optical WGMs having the same momentum separation. There also exist an extended group of acoustic bulk modes having the same *M* (representative members illustrated) that are also phase matched but are not observable above the noise floor due to high mechanical damping and poorer frequency matching. These are considered in our model collectively as a quasi-mode (*b*_±_)

The cylindrical symmetry of our system means that the whispering-gallery modes (WGMs) take the functional form *f*(*r*, *z*)e^*i*(*Mϕ*−*ωt*)^, where *ω* is the eigenfrequency and signed integer *M* describes the propagation momentum or azimuthal order on the angular spatial variable *ϕ*. The transverse mode profile is given by *f*(*r*, *z*). We now introduce the specific mechanical and optical WGMs that participate in our experiment, and the nature of their interaction. Our structure hosts frequency-adjacent optical modes belonging to different families that may be populated with photons of either pseudo-spin (Fig. 1a), for a total of four optical modes in the experiment. Scattering between these optical modes is only permitted through acousto-optic coupling (Brillouin scattering) via propagating phonons that match their frequency and momentum difference^29^, which is termed the phase matching requirement. Thus, photon modes having cw (ccw) pseudo-spin can only be coupled through scattering from co-propagating phonons having cw (ccw) pseudo-spin. In this situation when we pump the lower-energy optical mode, anti-Stokes scattering to the higher mode annihilates phonons^26^ of matched parity, resulting in added optomechanical damping and cooling of the phonon mode. This system should thus exhibit significant optically induced chirality in the transport of phonons that are phase matched for this interaction (Fig. 1a, b). A large family of mechanical WGMs satisfy these phase matching requirements, of which a few representative members are illustrated in Fig. 1c. The lowest transverse-order Rayleigh phonon mode is most likely to be observable as it interacts least with the supporting stem of the microsphere, i.e. features the highest *Q*-factor, and thus generates the strongest scattering between the optical modes^29^. However, disorder-induced scattering can couple this high-*Q* mode to the large population of lower-*Q* bulk modes of the resonator, as they have the same azimuthal order *M* but differ in extension into the bulk. Since the transverse optical mode profiles are much smaller than the transverse profiles of the mechanical modes (Fig. 1c), the bare optical mode coupling to each of these many mechanical (physical) modes is of a similar order. Below, we collectively treat the remaining lower-*Q* bulk modes as a pair of phonon “quasi-modes” having large dissipation rate and fixed pseudo-spin. These quasi-modes act as a thermal bath for their parity-flipped high-*Q* mode, while also directly coupling to the light.

### Experimental demonstration of chiral cooling

**Fig. 2:**
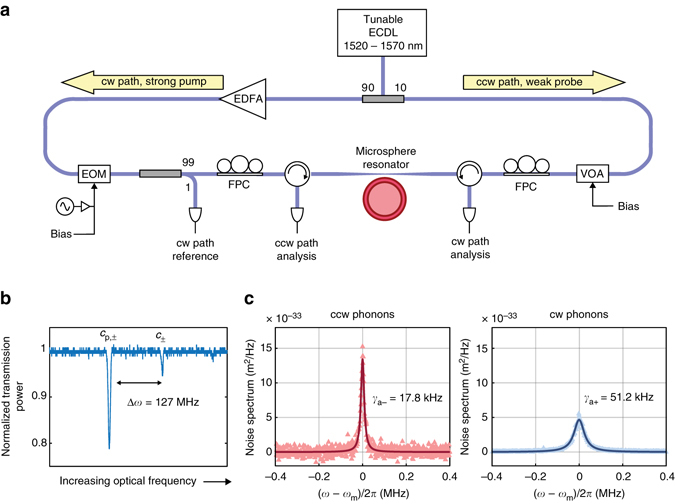
Measurement setup for chiral optomechanical refrigeration. **a** We perform the experiment using a silica whispering-gallery microsphere resonator that is interfaced via tapered optical fiber. A 1520 to 1570 nm tunable external cavity diode laser (ECDL) generates the asymmetric cw pump (strong) and ccw probe (weak) sources. An Erbium-doped fiber amplifier (EDFA) controls the cw pump power, for optomechanical cooling^26^ and for monitoring the cw phonon spectrum. An electro optic modulator (EOM) is employed to measure detuning of anti-Stokes scattered light from its optical mode through via induced transparency^19^. A variable optical attenuator (VOA) is used to control the ccw probe. Fiber polarization controllers (FPC) are used to optimize coupling between the fiber and resonator in both directions. **b** The optical modes are mapped by measuring dropped power from the tapered fiber. **c** Dramatic chiral cooling of the high-*Q* (*ω*_m_ = 2*π* × 127 MHz) phonon populations is immediately observed with strong cw pumping (140.9 μW). ccw phonons are also cooled slightly due to the ccw probe. Solid lines are Lorentzian fits to the data

Our experiments are performed with a silica WGR of diameter ~135 μm at room temperature and atmospheric pressure, using a tapered fiber coupler for optical interface at 1550 nm (Fig. 2a, b). The FWHM of both optical modes is approximately *κ* = 5.1 MHz, and the approximate simulated mode shapes are illustrated in Fig. 1c. Direct coupling between cw and ccw pseudo-spins, e.g., through optical Rayleigh scattering, is negligible for either optical mode in our experiment. The high-*Q* mechanical mode is also a whispering-gallery mode at 127 MHz with azimuthal order of *M* = 14 and mode shape illustrated in Fig. 1c. Verification of the Brillouin phase matching between these modes can be performed by means of forward Brillouin lasing^29^ and induced transparency measurements^19^. To examine the potential for modification of the high-*Q* phonon behavior, and the possibility of chiral transport of sound, we set up the experiment with two optical sources tuned to the lower optical mode in the clockwise (cw) and counter-clockwise (ccw) directions. The role of the stronger cw “pump” is to induce cooling of the cw propagating phonons, while the role of the much weaker ccw “reverse probe” is to measure, via optical scattering, the counter-propagating phonon behavior. The RF beat spectrum generated between the scattered light and the corresponding source in either direction provides a direct measure of the phonon mode spectrum (Supplementary Note 4), with sample measurements shown in Fig. 2c. In the experiment, the optical pump and probe sources are both derived from the same laser and are thus always at identical frequencies. Throughout the remainder of this work, no pump or probe field is delivered to the upper optical mode, in order to prevent coherent amplification of the phonons via Stokes Brillouin scattering.

Our first task is to measure the bare linewidth *γ*_m_ of the high-*Q* phonon mode without any optical pumping. We note that the bare linewidth (*γ*_m_) of high-*Q* mechanical WGMs may be qualitatively distributed into two forms of loss: those that maintain parity, which we call intrinsic dissipation (*γ*), and those that break parity, leading primarily to radiative damping via low-*Q* bulk modes. Thus *γ*_m_ is traditionally the minimum measurable linewidth in any optomechanical sideband cooling experiment. We perform this measurement by detuning the source laser from the optical resonance such that little to no optomechanical cooling is induced by either the pump or the probe. For zero input power, the bare linewidth *γ*_m_ = 12.5(±1.0) kHz is estimated by fit-based extrapolation of measured cw and ccw phonon linewidths *γ*_*a*+_ and *γ*_*a*−_ using the theoretical model (Supplementary Eqn. 5). We also obtain the single phonon optomechanical coupling strength *h*_0_ ≈ 14(±2.5) Hz. All uncertainties in this manuscript correspond to 95 % confidence bounds of the fitted value.

The optomechanical cooling rate can be controlled by the detuning *Δ*_2_ of the anti-Stokes scattered light from its optical mode, which we measure directly using the Brillouin Scattering Induced Transparency^19^. In Fig. 4a we plot measurements of both cw and ccw phonon linewidth as a function of this detuning. We immediately see a striking direction-dependence of the damping rates of the cw and ccw phonons, that has never previously been reported. This chiral damping of phonons is a direct result of the momentum conservation rules that underly the Brillouin scattering interaction, and will not generally be available in traditional single-mode optomechanical systems. We note also that the relative power of the cw pump and ccw probe lasers is ~9:1, so there is some sideband cooling of the *a*_−_ phonons as well.

**Fig. 3:**
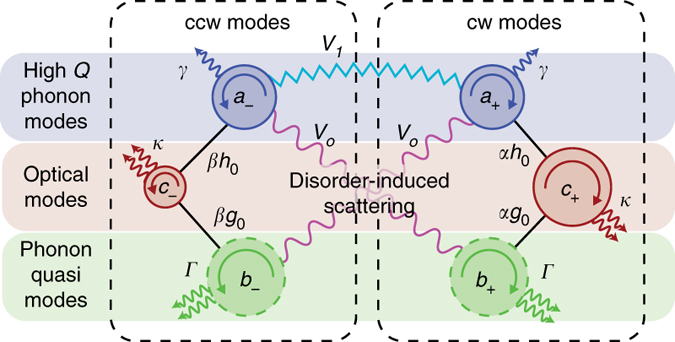
Model for coupling between the anti-Stokes (higher frequency) optical modes *c*_±_, high-*Q* phonon modes *a*_±_, and phonon quasi-modes *b*_±_ in cw and ccw directions. Light couples to the *a*_±_ and *b*_±_ phonons with different optomechanical interaction strength. Disorder-induced scattering between *a*_±_ phonons and the *b*_∓_ quasi-mode occurs with strength *V*_0_, and between *a*_±_ and *a*_∓_ high-*Q* phonons occurs with strength *V*_1_. Our experimental case features ${V_1} \ll {V_0}$. Details on individual parameters are provided in the text

### Model for chiral cooling

We now propose a model, detailed in ref. ^30^, that incorporates all the essential physics described above and illustrated in Fig. 3. Specifically, we define the higher frequency optical modes (anti-Stokes) through annihilation operators *c*_*σ*_, with parity *σ* = + for cw photons and *σ* = − for ccw photons. These optical modes couple to the matched high-*Q* phonon mode *a*_*σ*_, via direct optomechanical interaction with strength *αh*_0_ (or *βh*_0_), and also couple to the quasi-mode *b*_*σ*_ with strength *αg*_0_ (or *βg*_0_). In all cases these interactions conserve parity *σ*. *h*_0_ and *g*_0_ are the bare (single photon/phonon) optomechanical coupling strengths, while *α* and *β* are the square root of the intracavity photon number in cw and ccw direction respectively due to the pump and probe lasers. We note that the optomechanical coupling to the quasi-mode (itself comprised of many low-*Q* modes) can be large, due to the mode overlap highlighted in Fig. 1.

**Fig. 4:**
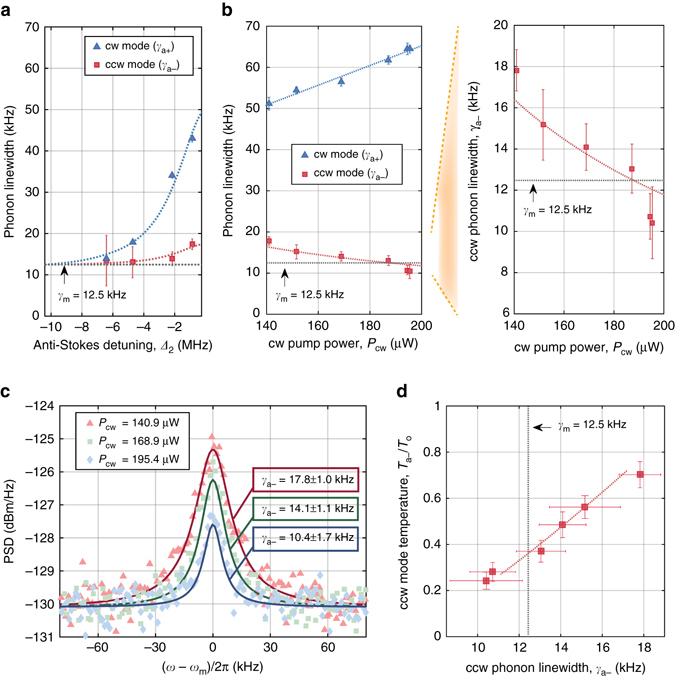
Chiral refrigeration in a silica whispering-gallery resonator. **a** Experimental measurement of bare phonon linewidth *γ*_m_ and observation of chiral asymmetry in dissipation rates for cw and ccw phonons during the initial sideband cooling experiment. Blue and red dashed lines in subfigures a and b are fits to theory in Supplementary Equation 5. **b** Observation of increasing ccw phonon coherence during sideband cooling of the cw propagating phonons (fixed ccw probe). The ccw phonon linewidth ${\gamma _{a - }}$ drops below the bare linewidth *γ*_m_, indicating reduction in the disorder-induced dissipation. **c** Measured photocurrent PSD (proportional to ccw phonon PSD) during experiment of subfigure b. The ccw phonons experience gain-free spectral narrowing and cooling (reduction in spectrum area) when the cw laser *P*_cw_ is increased. Solid lines are Lorentzian fits to the data. **d** Measured ccw phonon mode temperature $T_{{a_ - }}^{{\rm{eff}}}$ vs linewidth ${\gamma _{a - }}$ showing that, in contrast to sideband cooling, both the linewidth and temperature decrease during this experiment. Temperature is calculated using the integrated phonon power spectrum (examples in subfigure **c**), with vertical error bars generated from amplitude uncertainty. Red dashed line is a fit to the theory of equations (2) and (3). The phonon mode starts pre-cooled due to the sideband cooling from the probe. All error bars correspond to 95% confidence intervals for their respective parameters

To see the role chirality plays, we now also include the existence of disorder-induced scattering between *a*_*σ*_ and ${b_{\bar \sigma }}$ modes having strength *V*_0_—a term that explicitly breaks the conservation of parity. We note that the definition of our modes already account for low-angle scattering that conserves parity but leads to damping. Thus we do not include a direct *a*_*σ*_ to *b*_*σ*_ term in our theory, as it is included in the definition of intrinsic linewidths *γ* (for *a*_*σ*_) and *Γ* (for *b*_*σ*_). We can thus represent this toy model system with the interaction Hamiltonian expression given by:

1 $$\begin{array}{ccccc}\\ {H_{{\rm{int}}}} = & \alpha c_ + ^\dag \left( {{h_0}{a_ + } + {g_0}{b_ + }} \right) + \beta c_ - ^\dag \left( {{h_0}{a_ - } + {g_0}{b_ - }} \right)\\ \\ & + {V_0}\left( {a_ - ^\dag {b_ + } + b_ + ^\dag {a_ - }} \right) + {\rm{h}}{\rm{.c}}{\rm{.}}\\ \end{array}$$

The full model including the dissipation and detuning terms is provided in the Supplementary Information.

We can now derive the equations of motion for this system by means of the Heisenberg-Langevin equation (Supplementary Note 2). The key features of our data can be understood simply by a series of adiabatic elimination steps. First, we adiabatically eliminate the *c*_+_ optical mode with linewidth $\kappa \gg {\gamma _{\rm m}}$, which leads to sideband cooling of both *a*_+_ and *b*_+_. In particular, the mode *b*_+_ having bare linewidth *Γ*, is damped optically with a rate ${\it{\Gamma}} {{\cal C}_\alpha }$ from this adiabatic elimination. The parameter ${{\cal C}_\alpha }$ is the quasi-mode optomechanical cooperativity defined as $4{\alpha ^2}g_0^2{\rm{/}} {\it{\Gamma}} \kappa$.

Viewing the quasi-mode as a bath, we see that this cw bath is cooled to a temperature *T*_*b*+_ ≈ *T*_bulk_/($1+{{\cal C}_\alpha }$) via sideband cooling. This chiral refrigeration in turn modifies the damping and temperature of the *a*_−_ mode. Specifically, adiabatic elimination of the quasi-mode *b*_+_ leads to an effective damping of the *a*_−_ mode. At zero optical power, we have bare linewidth *γ*_m_ = *γ* + 4|*V*_0_|^2^/*Γ*, where *γ* is the intrinsic linewidth of *a*_−_. As we increase the optical power, we see that the disorder-induced damping term reduces due to the increased damping of the *b*_+_ quasi-mode—this is an optically induced impedance mismatch. Consequently, if there were no probe light, we would see that the *a*_−_ damping rate reduces to

2 $${\gamma _{{a_ - }}} = \gamma + \frac{{4{{\left| {{V_0}} \right|}^2}}}{{ {\it{\Gamma}} \left( {1 + {{\cal C}_\alpha }} \right)}} .$$

We can also see that the temperature of the *a*_−_ mode should go to a weighted sum of these two terms (details in Supplementary Note 2):

3 $${T_{a - }} = \frac{{\gamma {T_{{\rm{bulk}}}} + \left( {{\gamma _{{a_ - }}} - \gamma } \right){T_{b + }}}}{{{\gamma _{{a_ - }}}}}$$

As *T*_*b*+_ < *T*_bulk_, we see that for moderate ${{\cal C}_\alpha }$, the temperature *T*_*a*−_ goes down_,_ even as ${\gamma _{{a_ - }}} < {\gamma _{\rm m}}$! Conventional optomechanical cooling involves an increase of mechanical damping while the heat load remains constant, resulting in a lowering of the mode temperature. Here we see that while the damping reduces, the heat load on the system also reduces. This leads to a lower effective temperature of the mechanical system even as the linewidth narrows. Important corrections due to the finite probe power lead to additional broadening and cooling of the *a*_−_ mode, while the *a*_+_ mode’s dynamics are dominated by the sideband cooling from the *αh*_0_ coupling. But the key features are described by the above picture of chiral refrigeration.

We excluded a simpler model, of two degenerate mechanical modes *a*_±_ coupled by disorder of strength *V*_1_ and no additional quasi-modes, as it fails to produce two key features of the data below. First, at low pump power, we would see significant mode splitting (below we set an experimental bound for the direct coupling rate between *a*_±_ modes at *V*_1_ < 1 kHz), representing a breaking of parity conservation due to disorder-induced scattering. Second, at high pump powers, explored below, the smallest linewidth that the backward mode could achieve would be equivalent to its initial linewidth and its temperature would be equal to the bath temperature. We present a more detailed analysis of this model in Supplementary Note 2. Optical coupling to multiple (bulk) mechanical modes is the next best alternative, and as we show above, describes these phenomena.

### Disorder suppression and optomechanical cooling without damping

We now return to the experimental results to demonstrate the key predictions of this model: (i) damping associated with disorder-induced scattering can be optically inhibited, (ii) the damping rate of ccw phonons can be brought below the bare linewidth *γ*_m_, and (iii) the process leads to a reduction in heat load. Here, we employ an erbium-doped fiber amplifier to control the cw pump power (*P*_cw_) while keeping the ccw probe power constant at 12.5 μW. The anti-Stokes Brillouin scattered light in the resonator is kept close to zero detuning from its optical mode to maximize cooling efficiency, i.e., |*Δ*_2_/*κ*| is always less than 10%. Since the ccw probe adds some fixed optical damping to the ccw phonons, the initial measurement of ${\gamma _{a - }}$ is at 17.8(±1) kHz, which is greater than the bare linewidth γ_m_.

As we increase cw pump power from 140.9 to 195.4 *μ*W, the added optical damping broadens the cw phonon linewidth ${\gamma _{a + }}$ (Fig. 4b). The striking feature of this experiment is that the ccw phonon linewidth ${\gamma _{a - }}$ simultaneously reduces, i.e. the ccw phonons become more coherent! We verify that the increased coherence of the ccw phonons is not associated with any gain (Fig. 4c) by observing their spectrum through the measured photocurrent (Supplementary Note 4). In fact, quite the opposite occurs, and the total integrated area under the phonon spectrum also reduces, indicating a reduction in temperature of the ${a_ - }$ phonons. Since the ccw optical probe was not modified, the optically induced damping from the probe laser remains fixed. The reduction of the ${\gamma _{a - }}$ linewidth thus indicates that a hidden contribution to dissipation is being eliminated when the cw pump power is increased. At the highest power, the smallest dressed linewidth *γ*_*a*−_ = 10.5(±1.7) kHz is below the bare linewidth *γ*_m_ = 12.5(±1) kHz measured at the start of the experiment, even including the extra sideband damping from probe *β*. Fitting of the power vs linewidth measurements to our model (Supplementary Eqn. 5) reveals the ratio of coupling rates *V*_0_/*g*_0_ = 1.15(±0.05) × 10^3^. Our model indicates that the observed reduction in the phonon linewidth ${\gamma _{a - }}$ occurs due to reduction of the disorder-induced scattering. Specifically, the ccw propagating phonons achieve appreciable robustness against disorder due to chiral optomechanical damping of the cw phonon quasi-mode.

In Fig. 4d we present the temperature *T*_*a*−_ of the ccw phonon mode measured through phonon power spectral area, as a function of its measured linewidth. Fitting this temperature data to our model in Supplementary Note 2 permits extraction of $V_0^2{\rm{/}} {\it{\Gamma}} = 2.8( \pm 0.32)$ kHz. The parameters *V*_0_, *g*_0_, and *Γ* cannot presently be further separated since the phonon quasi-modes *b*_±_ are not directly observable. However, we note that the minimum self-consistent quasi-mode linewidth is approximately the optical linewidth (*Γ* ≈ *κ*) and we can obtain the values *V*_0_ = 121(±13) kHz and *g*_0_ = 105(±12) Hz at this minimum. These estimates are commensurate with our earlier assumption that the disorder-induced scattering between the high-*Q* and quasi-modes dominates over direct scattering between the high-*Q* modes (i.e. ${V_0} \gg {V_1}$). We additionally learn that the lower limit of *g*_0_ is roughly 7.5 times *h*_0_, implying that the optomechanical coupling to the quasi-mode is significant, and which agrees with the number of phonon modes that are likely to compose the quasi-mode. The anomalous cooling that we observe is thus well explained by significant coupling to, and chiral refrigeration of the cw quasi-mode bath.

## Discussion

Sideband cooling has been to date the only mechanism available for suppressing the thermal motion of mechanical resonators using light—but is necessarily accompanied by linewidth broadening. In this work, we have demonstrated the existence of a fundamentally different mechanism for cooling mechanical oscillators, that occurs through sideband cooling of the bath modes. No previous experiment in optomechanics has provided either direct or indirect evidence of such bath cooling. More importantly, this mechanism has the potential to revolutionize the noise calculus that we employ, since the cooling is instead accompanied by linewidth narrowing! In addition, we have demonstrated for the first time that not only can phonon chirality be induced optically, but also that it mitigates the influence of disorder on propagating phonons, a technique that potentially revolutionizes phonon-assisted measurements. To date such scattering immunity for phonons has only been demonstrated in topological insulators. Our results thus dramatically push forward the known physics for both laser cooling and for monolithic chiral systems.

Our approach for inducing chiral behavior is, at present, confined to the narrowband response of a high-*Q* resonator system. However, such devices are already in use for metrological applications^8–11^ including atomic force microscopes^31^ and quantum-regime transducers^32, 33^. In all these cases, increasing the quality factor while reducing the heat load of the mechanical element would lead to a direct improvement in performance. Furthermore, the modification of phonon transport by light may have substantial impact even beyond contemporary devices, as the ability to dynamically reconfigure the phononic behavior may change the realm of possibility as currently conceived. Still, robust demonstration of chiral asymmetry and nonreciprocal behavior remains close, and our work provides a foundation upon which to build such demonstrations.

### Data availability

Data can be made available by request to the authors on an individual basis.

## Electronic supplementary material

Supplementary Information
