# Supplementary material for: Dynamically induced robust phonon transport and chiral cooling in an optomechanical system
Source: Nat Commun. 2017 Aug 7;8:205. doi: 10.1038/s41467-017-00247-7 (PMC5547168; doi:10.1038/s41467-017-00247-7)
Supplement: Supplementary file 1 — Supplementary Information [file 41467_2017_247_MOESM1_ESM.pdf]

Title: Supplementary Information

Description: Supplementary Table, Supplementary Notes, Supplementary Figures, and Supplementary References

Supplementary Table 1. Table of Symbols

symbol	meaning
$c_{p,\sigma}$, c_σ	Two frequency-adjacent optical modes; the pump mode ($c_{p,\sigma}$) and the anti-Stokes mode (c_σ)
$c_{+(-)}$	Annihilation operator of the optical mode in the cw(ccw) direction
$a_{+(-)}$	Annihilation operator of the high-Q phonon mode in the cw(ccw) direction
b_k	Annihilation operator of the phonon modes in the system
λ_k	Optomechanical single-photon coupling strengths to the two optical modes; the c_σ and $c_{p,\sigma}$ modes
Λ_k	Pump-enhanced optomechanical coupling constant, $\Lambda_k \triangleq \lvert\alpha\lambda_k\rvert$.
μ_k	Coupling strength of disorder-induced scattering between the $a_\pm$ modes to the b_k modes
$b_{+(-)}$	Annihilation operator of the phonon quasi-mode in the cw(ccw) direction
$c^{\rm in}_{+(-)}$	Annihilation operator of the optical noise in the $c_{+(-)}$ modes
$a^{\rm in}_-$, $b^{\rm in}_+$	Annihilation operator of the thermal noise in the a_- and b_+ modes, respectively
$a^{\rm eff}_-$	Annihilation operator of the effective thermal noise in the a_- mode
h_0, g_0	Optomechanical single-photon coupling strengths, $h_0^2 + g_0^2 = 1$
V_0	Coupling strength of disorder-induced scattering between the $a_\pm$ modes to the $b_\mp$ mode
ω_1	Cavity resonance frequency of the pump optical mode
ω_2	Cavity resonance frequency of the anti-Stokes optical mode
ω_L	Pump laser frequency
ω_m	Mechanical resonance frequency
δ	Detuning of the pump mode from the pump laser, $\delta = \omega_1 - \omega_L$
$\varDelta$	Detuning of the anti-Stokes mode from the pump laser, $\varDelta = \omega_2 - \omega_L$
$\varDelta_2$	Detuning of the anti-Stokes mode from the scattered light, $\varDelta_2 = \omega_2 - (\omega_L + \omega_m)$
κ_0	Intrinsic loss rate of the anti-Stokes optical mode
κ_{ex}	Loss rate associated with the external coupling
κ	Measurable optical linewidth of the anti-Stokes mode, $\kappa = \kappa_0 + \kappa_{ex}$
$\kappa_{\rm p}$	Measurable optical linewidth of the pump mode
γ	Mechanical damping rate of the high-Q phonon modes
$\varGamma$	Mechanical damping rate of the phonon quasi-modes
$n_+ = \lvert\alpha\rvert^2$	Intracavity photon number in the cw optical mode c_+
$n_- = \lvert\beta\rvert^2$	Intracavity photon number in the ccw optical mode c_-
$\mathcal{C}_\alpha$	Cooperativity of the c_+ mode, $\mathcal{C}_\alpha = 4\alpha^2 g_0^2/\varGamma\kappa$
$\mathcal{C}_\beta$	Cooperativity of the c_- mode, $\mathcal{C}_\beta = 4\beta^2 g_0^2/\varGamma\kappa$
$\chi^{-1}_{a_\pm}(\omega)$	Mechanical susceptibilities of the $a_\pm$ modes
$T_{a_\pm}$	Bath temperatures of the $a_\pm$ modes
$T^{\rm eff}_{a_\pm}$	Effective temperatures of the $a_\pm$ modes
n_L	Intracavity photon number in the pump optical mode driven by the pump laser
$\bar{n}$	Effective phonon occupation number of the a_- phonon mode
$S_{a_-}(\omega)$	Quantum noise spectrum of a_- mode, $S_{a_-}(\omega) = \int_{-\infty}^{\infty} dte^{i\omega t}\langle a_-(t)a_-(0)\rangle$
$S_{II}(\omega)$	Quantum noise spectrum of normalized photocurrent $I(t)$, $S_{II}(\omega) = \int_{-\infty}^{\infty} dte^{i\omega t}\langle I(t)I(0)\rangle$

Supplementary Note 1. Defining the modes and their coupling

Our system is composed of cw and ccw optical modes, high-Q phonon modes, as well as vibrational excitations inside the material, i.e. the phonon bath. As described in the main text, we focus on a scenario in which high-Q modes of clockwise and counterclockwise circulation with annihilation operators a_σ are coupled via disorder to a quasi-mode (broad mode representing many actual mechanical modes) circulating in the opposite direction with annihilation operators $b_{\bar{\sigma}}$. In the experiment, for each circulation we have a pair of optical modes $c_{p,\sigma}$ and c_σ. The optomechanical coupling allows for transfer of light from $c_{p,\sigma}$ to c_σ with a corresponding annihilation of a phonon that is phase matched. This process overlaps with both the high-Q modes and the quasi-modes.

With the above basic picture, we examine this model using the rotating wave approximation (RWA) as the experimental configuration is all narrowband. We can then use the input-operator language to describe the open system dynamics. After displacing the optical cavity fields by the pump amplitudes in the cavity, $c_\sigma \to \sqrt{n_\sigma}+c_\sigma$ with $\sqrt{n_+} = \alpha$, $\sqrt{n_-} = \beta$, the $c_{p,\sigma}$ fluctuations decouple from the rest of the system. Working in the frame rotating with the pump laser frequency, we write the linearized Heisenberg-Langevin equations for the mechanical and optical modes in the Fourier domain with Fourier frequency ω:

$$-i\omega c_\sigma = -i\Delta c_\sigma - \frac{\kappa}{2}c_\sigma + \sqrt{\kappa}c_\sigma^{\text{in}} - i\sqrt{n_\sigma}(g_0 b_\sigma + h_0 a_\sigma) \tag{1a}$$

$$-i\omega a_\sigma = -i\omega_m a_\sigma - \frac{\gamma}{2}a_\sigma + \sqrt{\gamma}a_\sigma^{\text{in}} - iV_0 b_{\bar{\sigma}} - ih_0\sqrt{n_\sigma}c_\sigma \tag{1b}$$

$$-i\omega b_\sigma = -i\omega_b b_\sigma - \frac{\Gamma}{2}b_\sigma + \sqrt{\Gamma}b_\sigma^{\text{in}} - i\alpha h_0 c_\sigma - iV_0 a_{\bar{\sigma}} \tag{1c}$$

where V_0 is the coupling strength of disorder-induced scattering between the cw(ccw) high-Q phonon modes a_σ to the ccw(cw) phonon quasi-modes $b_{\bar{\sigma}}$. In contrast to the usual quantum optics literature, we here define detuning to be the mode frequency minus the signal frequency. Thus a positive detuning is red detuned. This makes comparison to mechanical motion as transparent as possible. Meanwhile, the coupling of the quasi-modes b_σ to the c_σ modes is given by $g_0\sqrt{n_\sigma}$, while $h_0\sqrt{n_\sigma}$ captures the coupling between a_σ and c_σ. For any given ω, there is a self-consistent $\omega_b \approx \omega$ that describes the relevant portion of the bath modes. Thus we take $|\omega_b - \omega| \ll \Gamma$ in what follows. Specifically, we have the following main assumptions for this simple model:

1. Phonon backscattering occurs between high-Q phonon modes and the phonon quasi-modes, i.e. between $a_+ \longleftrightarrow b_-$ and $a_- \longleftrightarrow b_+$, with strength V_0.

2. The cw(ccw) optical mode $c_{+(-)}$ couples to the high-Q phonon mode $a_{+(-)}$ and the cw(ccw) phonon quasi-mode $b_{+(-)}$ with different strengths. The cw optical mode c_+ couples to the cw high-Q mode a_+ via direct optomechanical interaction with strength αh_0 and couples to the quasi-mode with strength αg_0. Likewise, the ccw

optical mode c_- couples to the ccw high-Q mode a_- with strength βh_0 and couples to the ccw quasi-mode b_- with strength βg_0. Here are n_+ and n_- are the number of intracavity photon in the cw optical mode c_+ and the ccw mode c_-, respectively.

3. The high-Q phonon modes $a_{+(-)}$ and the phonon quasi-modes $b_{+(-)}$ have the intrinsic damping rates γ and Γ, respectively ($\gamma \ll \Gamma$). The cw modes and ccw modes have symmetry with respect to the origin. We also assume that the damping rate Γ is in the same order as the optical loss rate κ.

We exclude a simpler model, of two degenerate mechanical modes and no additional quasi-modes, as it fails to produce two key features of the data. First, at low pump power, we would experimentally observe some mode splitting, representing a breaking of circular symmetry from disorder-induced scattering. Second, at high pump power, the lowest linewidth the backward mode could achieve would be equivalent to its initial linewidth, and its temperature would be equal to the bath temperature. Optical coupling to multiple mechanical modes is the next best alternative, and as we show here, describes these phenomena.

Based on these assumptions, we can obtain the simplified continuum model as shown in Supplementary Fig. 1.**a**. In principle, the dynamics of the system can be solved numerically. However the loop structure in this coupled six-mode system will complicate the result, rendering interpretation difficult. To better capture the main physics, we can make the following approximation: we assume the g_0 parameter is larger than h_0 so that the optical field couples more strongly to the bulk modes $b_\pm$. We note that this assumption is not actually that important for our main result, as the crucial point is that the dominant mechanical damping mechanism is coupling of the high-Q modes to the quasi-modes – this is independent of g_0 and h_0. We can then break the loop into two pieces (see Supplementary Fig. 1.**a**-**c**).

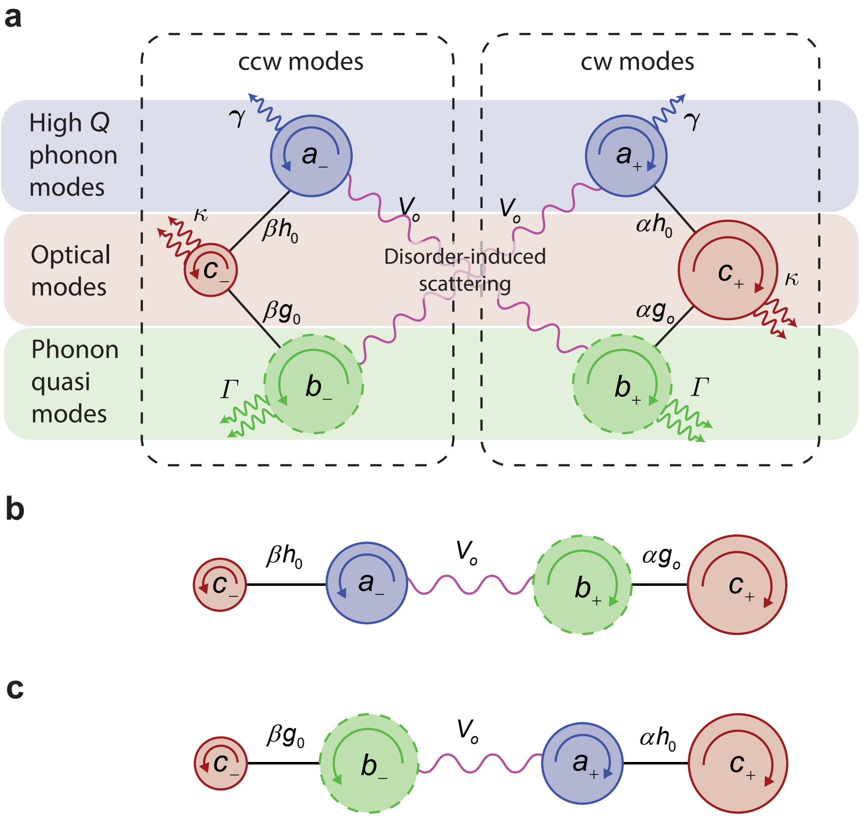

Supplementary Figure 1: (a) Full description of the optomechanical coupling of the $c_\pm$ modes to $a_\pm$ modes and $b_\pm$ modes, and the disorder-induced scattering between the $a_\pm$ modes and $b_\mp$ modes. Separated optomechanical coupling descriptions by coupling directions (b)-(c). (b) The ccw-side coupling that the optical mode c_- is mainly coupled to the a_- mode with the disorder-induced scattering. (c) Likewise, the cw-side coupling that the cw mode c_- is coupled to the a_+ mode.

Supplementary Note 2. Analysis of the simplified continuum model

SN2.1 Susceptibilities of the high-Q modes, $a_\pm$

We first focus on the ccw-side coupling direction (Supplementary Figure 1.**b**) to calculate the linewidth of the ccw phonon a_-. Solving in the Fourier domain, we get

$$\begin{aligned}&\left[-i(\omega-\omega_m)+\gamma/2+\frac{\beta^2 h_0^2}{-i(\omega-\Delta)+\kappa/2}+\frac{V_0^2}{\Gamma/2}\left(1-\frac{\alpha^2 g_0^2}{\Gamma\tilde{\kappa}/2}\right)\right]a_- \\ =\ & \sqrt{\gamma}a_-^{\text{in}}-\frac{i\beta h_0\sqrt{\kappa}}{-i(\omega-\Delta)+\kappa/2}c_-^{\text{in}}-\frac{V_0\alpha g_0\sqrt{\kappa}}{\Gamma\tilde{\kappa}/2}c_+^{\text{in}}-\frac{iV_0}{\sqrt{\Gamma}/2}\left(1-\frac{\alpha^2 g_0^2}{\Gamma\tilde{\kappa}/2}\right)b_+^{\text{in}} \qquad (2)\end{aligned}$$

where $\tilde{\kappa}\triangleq -i(\omega-\Delta)+\kappa/2+2\alpha^2 g_0^2/\Gamma$. Similarly, for the cw phonon mode a_+, we can find its equation of motion by interchanging α with β, a_+ with a_-, and c_+ with c_-:

$$\begin{aligned}&\left[-i(\omega-\omega_m)+\gamma/2+\frac{\alpha^2 h_0^2}{-i(\omega-\Delta)+\kappa/2}+\frac{V_0^2}{\Gamma/2}\left(1-\frac{\beta^2 g_0^2}{\Gamma\tilde{\kappa}'/2}\right)\right]a_+ \\ =\ & \sqrt{\gamma}a_+^{\text{in}}-\frac{i\alpha h_0\sqrt{\kappa}}{-i(\omega-\Delta)+\kappa/2}c_+^{\text{in}}-\frac{V_0\beta g_0\sqrt{\kappa}}{\Gamma\tilde{\kappa}'/2}c_-^{\text{in}}-\frac{iV_0}{\sqrt{\Gamma}/2}\left(1-\frac{\beta^2 g_0^2}{\Gamma\tilde{\kappa}'/2}\right)b_-^{\text{in}} \qquad (3)\end{aligned}$$

with $\tilde{\kappa}'\triangleq -i(\omega-\Delta)+\kappa/2+2\beta^2 g_0^2/\Gamma$. The susceptibilities of the $a_\pm$ modes are given by the left hand side of the equations of motion:

$$\chi_{a_+}^{-1}(\omega) = -i(\omega-\omega_m)+\gamma/2+\frac{\alpha^2 h_0^2}{-i(\omega-\Delta)+\kappa/2}+\frac{V_0^2}{\Gamma/2}\left(1-\frac{\beta^2 g_0^2}{\Gamma\tilde{\kappa}'/2}\right), \qquad (4a)$$

$$\chi_{a_-}^{-1}(\omega) = -i(\omega-\omega_m)+\gamma/2+\frac{\beta^2 h_0^2}{-i(\omega-\Delta)+\kappa/2}+\frac{V_0^2}{\Gamma/2}\left(1-\frac{\alpha^2 g_0^2}{\Gamma\tilde{\kappa}/2}\right). \qquad (4b)$$

SN2.2 Linewidths of the $a_\pm$ modes

We can define the cooperativities as $\mathcal{C}_\alpha = 4\alpha^2 g_0^2/\Gamma\kappa$ and $\mathcal{C}_\beta = 4\beta^2 g_0^2/\Gamma\kappa$, which are both dimensionless parameters describing the strength of optomechanical coupling relative to cavity decay rate and mechanical damping rate. Under the phase matching condition that the pump laser and its scattered light are near the two frequency-adjacent optical modes, we can expect $\Delta \approx \omega_m$ (see Supplementary Fig. 2). To evaluate the linewidth of the high-Q phonon modes, we set $\omega \approx \omega_m$. Then we have:

$$\gamma_{a_+} = \gamma+\frac{4\alpha^2 h_0^2}{\kappa}+\frac{4V_0^2}{\Gamma}\frac{\kappa}{\kappa+4\beta^2 g_0^2/\Gamma}=\gamma+\frac{4\alpha^2 h_0^2}{\kappa}+\frac{4V_0^2}{\Gamma}\frac{1}{1+\mathcal{C}_\beta}, \qquad (5a)$$

$$\gamma_{a_-} = \gamma + \frac{4\beta^2 h_0^2}{\kappa} + \frac{4V_0^2}{\Gamma}\frac{\kappa}{\kappa + 4\alpha^2 g_0^2/\Gamma} = \gamma + \frac{4\beta^2 h_0^2}{\kappa} + \frac{4V_0^2}{\Gamma}\frac{1}{1+\mathcal{C}_\alpha} \tag{5b}$$

where γ_{a_+} and γ_{a_-} are the linewidths of the high-Q phonon modes $a_\pm$. Note that the linewidths $\gamma_{a_\pm}$ are larger than their minimum measurable linewidth $\gamma_m = \gamma + \frac{4V_0^2}{\Gamma}$ (obtained when optical power is zero, i.e. $\alpha = 0$, $\beta = 0$) due to the disorder induced backscattering to the counter-propagating quasimode (see Eq. 2)

SN2.3 Effective temperature of the phonon modes

Another important feature that comes from the continuum model is the reduction in the effective temperature of the a_- mode, because of coherent damping of the $\sigma = +$ mechanical modes. When the right-hand side of the equation (2) is considered with an assumption that the optical noise c_σ^{in} is negligible compared to the thermal noise source, we have the effective noise on a_- as:

$$\sqrt{\gamma}a_-^{\mathrm{in}} - \frac{iV_0}{\sqrt{\Gamma}/2}\left(1 - \frac{\alpha^2 g_0^2}{\Gamma\tilde{\kappa}/2}\right) b_+^{\mathrm{in}} \tag{6}$$

The effective temperature of mode a_- is then given by:

$$\begin{aligned} T_{a_-}^{\mathrm{eff}} &= \frac{1}{\gamma_{a_-}}\left[\gamma + \frac{V_0^2}{\Gamma/4}\left|1 - \frac{\alpha^2 g_0^2}{\Gamma\tilde{\kappa}/2}\right|^2\right] T_{\mathrm{bulk}} \\ &= \frac{1}{\gamma_{a_-}}\left[\gamma + \frac{4V_0^2}{\Gamma}\frac{(\omega - \Delta)^2 + \kappa^2/4}{(\omega-\Delta)^2 + (\kappa/2 + 2\alpha^2 g_0^2/\Gamma)^2}\right] T_{\mathrm{bulk}} \end{aligned} \tag{7}$$

When near resonance, $\omega \approx \Delta$, we have:

$$\begin{aligned} T_{a_-}^{\mathrm{eff}} &= \frac{1}{\gamma_{a_-}}\left[\gamma + \frac{4V_0^2}{\Gamma}\frac{\kappa^2/4}{(\kappa/2 + 2\alpha^2 g_0^2/\Gamma)^2}\right] T_{\mathrm{bulk}} \\ &= \frac{1}{\gamma_{a_-}}\left[\gamma + \frac{4V_0^2}{\Gamma}\frac{1}{(1+\mathcal{C}_\alpha)^2}\right] T_{\mathrm{bulk}} \end{aligned} \tag{8}$$

It reveals that the second term in equation (8) decreases with increasing $\mathcal{C}_\alpha$. This fact indicates the effective temperature of the ccw a_- mode reduces with increase of the cw pump laser ($\propto |\alpha|^2$). We can derive the effective temperature of the a_+ mode in the same manner.

$$T_{a_+}^{\mathrm{eff}} = \frac{1}{\gamma_{a_+}}\left[\gamma + \frac{4V_0^2}{\Gamma}\frac{1}{(1+\mathcal{C}_\beta)^2}\right] T_{\mathrm{bulk}}. \tag{9}$$

In the experiment shown in the main paper, the ccw probe β is much smaller compared to the cw pump α, thus this effective temperature $T_{a_+}^{\mathrm{eff}}$ change is not significant for the a_+ mode.

SN2.4 Analysis of the direct coupling model

For the direct coupling model, i.e. disorder only coupling a_- and a_+ via V_1 and no additional quasi-modes, we can find striking differences from the observations (See Supplementary Fig. 3). After adiabatic elimination of $c_\pm$, we have Heisenberg-Langevin equations for $V_0 = 0$ of

$$\dot{a}_- = -[i\omega_m + (\gamma + \mathit{\Gamma}_\beta)/2]a_- + \sqrt{\gamma}a_{-,in} - iV_1 a_+ \quad (10)$$

$$\dot{a}_+ = -[i\omega_m + (\gamma + \mathit{\Gamma}_\alpha)/2]a_+ + \sqrt{\gamma}a_{+,in} - iV_1 a_- \quad (11)$$

with $\mathit{\Gamma}_\alpha \equiv 4h_0^2|\alpha|^2/\kappa$ the optically-induced damping. We see that the normal modes of these equations have resonance conditions corresponding to two poles:

$$\omega_\pm = \omega - i\mathit{\Gamma}_\Sigma/2 \pm \sqrt{V_1^2 - \delta\mathit{\Gamma}^2/4} \quad (12)$$

where $\mathit{\Gamma}_\Sigma = \gamma + \frac{\mathit{\Gamma}_\beta + \mathit{\Gamma}_\alpha}{2}$ is the average damping and $\delta\mathit{\Gamma} = |\mathit{\Gamma}_\alpha - \mathit{\Gamma}_\beta|$ is the difference in damping. Thus at zero power the two poles are split on the real axis by $\pm V_1$, leading to mode splitting which is not observed in the experiment. Furthermore, as $\delta\mathit{\Gamma}$ increases to be larger than V_1, the damping rates start to differ, whereas in the experiment the damping is different for all optical powers. Finally, at high $\delta\mathit{\Gamma}$, the imaginary (damping) part of the pole is still always $\geq \gamma$, the value of the damping at zero optical power in this model, counter to the observed behavior in the experiment. Regarding the temperature of the a_- mode, working in the large $\delta\mathit{\Gamma}$ limit, we do see some cooling of a_- at intermediate powers, as predicted in Supplementary Ref. [1].

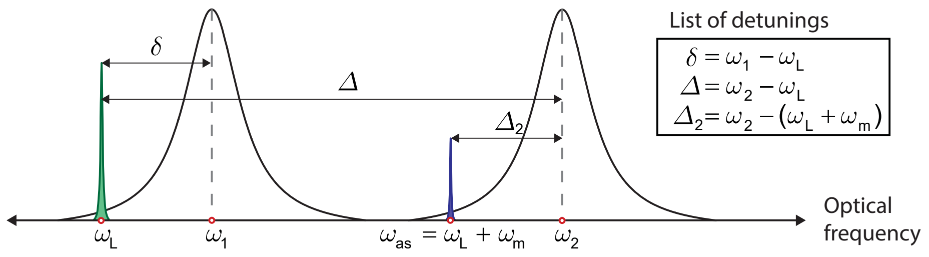

Supplementary Figure 2: Optical frequency relationship of the triplet resonant system for the experiment. The pump laser is at frequency ω_L, and the anti-Stokes scattered light appears at frequency $\omega_{as} = \omega_L + \omega_m$ via Brillouin scattering. The two frequency-adjacent optical modes are at ω_1 and ω_2, and the corresponding signal detunings are defined as $\delta = \omega_1 - \omega_L$, $\varDelta = \omega_2 - \omega_L$ and $\varDelta_2 = \omega_2 - \omega_{as}$.

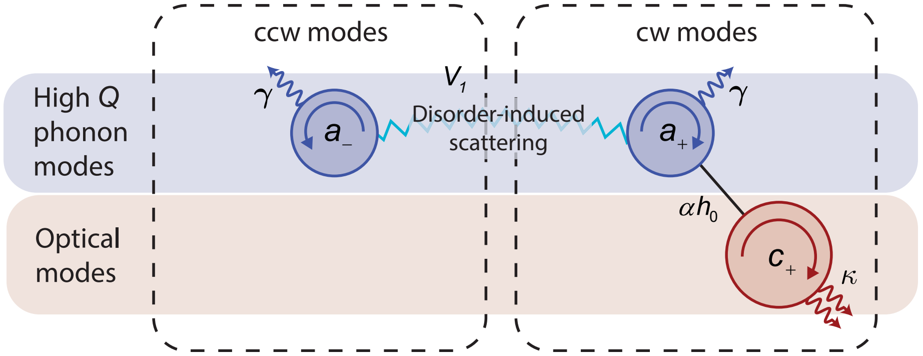

Supplementary Figure 3: The 'direct coupling model' consists of the optomechanical coupling of the c_+ optical mode to the a_+ high-Q mode, and direct disorder-induced coupling between the $a_\pm$ modes. αh_0 is the light-enhanced optomechanical coupling strength and V_1 is the direct coupling rate.

Supplementary Note 3. Experimental details

In Supplementary Fig. 4, we illustrate the detailed experimental setup used to measure the spectra of the cw (ccw) high-Q phonon modes $a_{\pm}$. The experiment is performed using a silica microsphere resonator optical $Q > 10^8$ that is evanescently coupled to a tapered fiber waveguide. The scattered light that results from the opto-acoustic coupling is sent to the photodetector through the same waveguide that also carries the pump laser. A tunable External Cavity Diode Laser (ECDL) spanning 1520 - 1570 nm drives light into the waveguide. A 90/10 fiber optic splitter separates this source into the cw pump and the ccw probe laser. In the cw direction, the pump laser is amplified by an Erbium-Doped Fiber Amplifier (EDFA). Thus, the EDFA affects the cw pump laser only, not the ccw probe laser.

In order to measure anti-Stokes light detuning from the optical mode, $\Delta_2 = \omega_2 - \omega_{AS}$, we employ a Brillouin Scattering Induced Transparency measurement [2]. Here ω_2 is the resonant frequency of the anti-Stokes optical mode, and ω_{AS} is the frequency of anti-Stokes scattered field via Brillouin scattering as illustrated in Supplementary Fig. 2. An electro-optic modulator (EOM) is used to generate the required probe sidebands relative to the cw pump laser. The upper sideband is used to probe the acousto-optic interference within anti-Stokes mode.

1 % of the signal after the EOM output is used as a reference to a network analyzer (NA) to measure the transfer function for this optical probe. The remaining light passes through a fiber polarization controller (FPC) to maximize coupling between the taper and the resonator. Circulators are employed for performing analysis of the cw and ccw scattered light. An oscilloscope (OSC) and a real-time spectrum analyzer (RSA) are used to measure these optical signals on a photodetector.

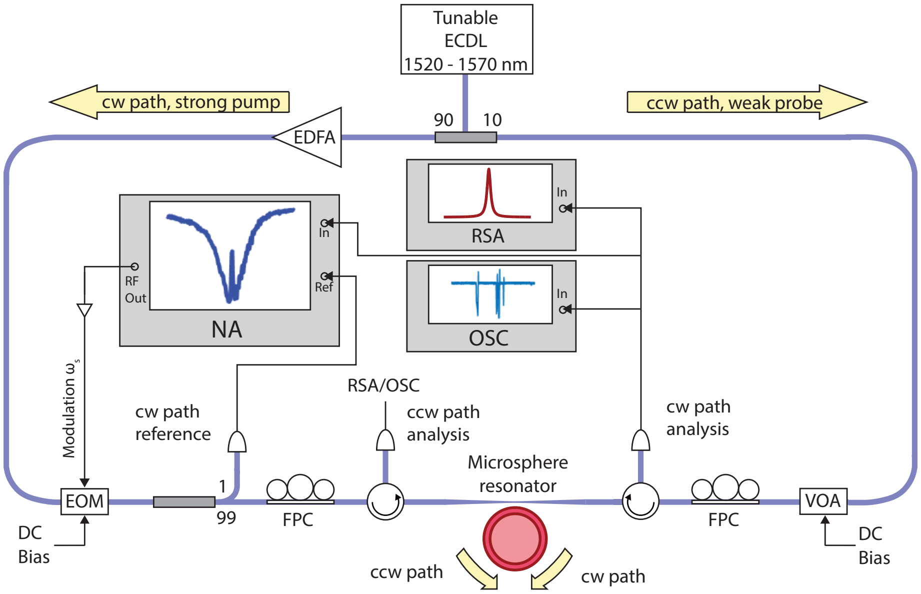

Supplementary Figure 4: Detailed experimental setup. The blue lines indicate the optical paths (fiber), while the black narrow lines indicate the electrical signal paths. A fiber-coupled tunable external cavity diode laser (ECDL) provides light through a 90/10 optical coupler that splits the light into cw and ccw directions. The cw pump power is controlled by an Erbium-doped fiber amplifier (EDFA). An electro optic modulator (EOM) is employed for probing detuning of anti-Stokes light from its optical mode. Scattered light is captured at a photodetector and is sent to an oscilloscope (OSC) and a real-time spectrum analyzer (RSA) for analysis.

Supplementary Note 4.
Measuring phonon mode spectra using a photodetector

To experimentally confirm the reduction of intrinsic damping of the high-Q phonon mode, we have to understand the measurement of the output spectrum at the photodetector. Using the equations (1, 6), we can rewrite the Heisenberg-Langevin equations for the a_- phonon mode in frequency domain with the non-depleted pump approximation:

$$-i\omega c_{p,\sigma} = -i\delta c_{p,\sigma} - \frac{\kappa_\mathrm{p}}{2} + \sqrt{\kappa_\mathrm{ex}}S, \tag{13a}$$

$$-i\omega c_- = -i\Delta c_- - \frac{\kappa}{2}c_- - i\beta h_0 a_- + \sqrt{\kappa_\mathrm{ex}}c_-^\mathrm{in}, \tag{13b}$$

$$-i\omega a_- = -i\omega_m a_- - \frac{\gamma}{2}a_- - i\beta h_0 c_- + \sqrt{\gamma}a_-^\mathrm{eff}, \tag{13c}$$

$$\text{where } \sqrt{\gamma}a_-^\mathrm{eff} = \sqrt{\gamma}a_-^\mathrm{in} - \frac{iV_0}{\sqrt{\Gamma}/2}\left(1 - \frac{\alpha^2 g_0^2}{\Gamma\tilde{\kappa}/2}\right) b_+^\mathrm{in}.$$

Here the stationary ergodic noise forces S, c_-^in and a_-^eff are the quantum Langevin noise of the $c_{p,\sigma}$, c_- and a_- modes, respectively. The quantum correlation functions of these noise forces are given by:

$$\begin{aligned}
\langle S^\dagger(t)S(t')\rangle &= n_L(t'-t),\\
\langle S(t)S^\dagger(t')\rangle &= (n_L+1)(t'-t),\\
\langle c_-^{\mathrm{in}\,\dagger}(t)c_-^\mathrm{in}(t')\rangle &= 0,\\
\langle c_-^\mathrm{in}(t)c_-^{\mathrm{in}\,\dagger}(t')\rangle &= \delta(t-t'),\\
\langle a_-^{\mathrm{eff}\,\dagger}(t)a_-^\mathrm{eff}(t')\rangle &= \bar{n}\delta(t-t'),\\
\langle a_-^\mathrm{eff}(t)a_-^{\mathrm{eff}\,\dagger}(t')\rangle &= (\bar{n}+1)\delta(t-t')
\end{aligned}$$

where n_L is the photon occupation number from the pump laser and $\bar{n}$ is the effective occupation number of phonons. We then obtain the noise spectrum of the a_- mode as follows:

$$a_-(\omega) = \frac{\sqrt{\gamma}a_-^\mathrm{eff}(\omega)}{\gamma_\mathrm{e}/2 + i(\omega_m - \omega)} - \frac{i\beta h_0\sqrt{\kappa_\mathrm{ex}}c_-^\mathrm{in}}{[\gamma_\mathrm{e}/2 + i(\omega_m-\omega)]\,[\kappa/2 - i(\omega - \Delta)]} \tag{14}$$

where ω_m is the effective mechanical frequency including the optical spring effect and $\gamma_\mathrm{e} = \gamma + \gamma_\mathrm{opt}$ is the effective mechanical damping rate including the optomechanical damping rate γ_opt. We can then derive the output spectrum measured at the downstream photodetector after the resonator. Using the input-output theory [3], we obtain the expression for the output field in the optical waveguide.

$$\begin{aligned}
S_{\rm out}(\omega) &= S_{in}(\omega) - \sqrt{\kappa_{\rm ex}}c(\omega) \\
&= S(\omega)\left[1 - \frac{2\kappa_{\rm ex}}{\kappa_{\rm p} - 2i(\omega-\delta)}\right] \\
&\quad + c_{-}^{\rm in}(\omega)\left[1 - \frac{2\kappa_{\rm ex}}{\kappa - 2i(\omega-\Delta)} + \frac{|\beta|^2|h_0|^2\kappa_{\rm ex}}{[\gamma_{\rm e}/2 + i(\omega_m-\omega)]\,[\kappa/2 - i(\omega-\Delta)]^2}\right] \\
&\quad + a_{-}^{\rm eff}(\omega)\frac{i\beta h_0\sqrt{\gamma}\sqrt{\kappa_{\rm ex}}}{[\gamma_{\rm e}/2 + i(\omega_m-\omega)]\,[\kappa/2 - i(\omega-\Delta)]} \\
&= s_1(\omega)S(\omega) + s_2(\omega)c_{-}^{\rm in}(\omega) + s_b(\omega)a_{-}^{\rm eff}(\omega)
\end{aligned} \tag{15}$$

where we are introducing the scattering matrix elements $s_1(\omega)$, $s_2(\omega)$ and $s_b(\omega)$ defined in [4]. The output spectrum at the photodetector is related to the spectrum of the normalized photocurrent $S_{II}(\omega')\delta(\omega-\omega') = \langle I(\omega)^\dagger I(\omega')\rangle$ where $I(\omega) = S_{\rm out}(\omega) + S_{\rm out}^\dagger(\omega')$. Thus, $S_{II}(\omega)$ is:

$$S_{II}(\omega) = \left(|s_1(\omega)|^2 + |s_2(\omega)|^2 + |s_b(\omega)|^2\right) + 2n_L|s_1(\omega)|^2 + 2\bar{n}|s_b(\omega)|^2 \tag{16}$$

The phonon noise spectrum $S_{a_-}(\omega) = \frac{\bar{n}\gamma}{(\gamma_e/2)^2 + (\omega_m-\omega)^2}$ is included in the above expression through the scattering element $2\bar{n}|s_b(\omega)|^2$, since $2\bar{n}|s_b(\omega)|^2 = \frac{2\gamma_{\rm opt}\kappa_{\rm ex}}{\kappa}\frac{\bar{n}\gamma}{(\gamma_e/2)^2+(\omega_m-\omega)^2}$. The remainder of the equation constitutes the noise floor N, which is a function of n_L. The resulting photocurrent spectrum is given by:

$$S_{II}(\omega) = N + \frac{2\gamma_{\rm opt}\kappa_{\rm ex}}{\kappa}S_{a_-}(\omega) \tag{17}$$

Thus, the measured RF output spectrum at the photodetector (ignoring noise floor N) is proportional to the spectrum of the high-Q phonon mode a_-, scaled by the optomechanical damping rate $\gamma_{\rm opt} = \frac{4|\beta|^2|h_0|^2}{\kappa}$ when $\omega \approx \omega_m$. Fixing the ccw probe power while measuring the spectrum of the ccw high-Q phonon mode a_- ensures that the magnitude scaling of the output spectrum is not affected by the ccw probe power. Thus, the spectrum obtained in $S_{II}(\omega)$ is directly representative of the phonon population and temperature of the mode.

Supplementary References

[1] X. Xu, T. Purdy, and J. M. Taylor, "Cooling a harmonic oscillator by optomechanical modification of its bath," *arXiv.org*, Aug 2016, arXiv:1608.05717.

[2] J. Kim, M. C. Kuzyk, K. Han, H. Wang, and G. Bahl, "Non-reciprocal Brillouin scattering induced transparency," *Nature Physics*, vol. 11, no. 3, pp. 275–280, Mar. 2015.

[3] C. Gardiner and P. Zoller, *Quantum noise: a handbook of Markovian and non-Markovian quantum stochastic methods with applications to quantum optics.* Springer Science & Business Media, 2004.

[4] A. H. Safavi-Naeini and O. Painter, "Proposal for an optomechanical traveling wave phonon-photon translator," *New Journal of Physics*, vol. 13, no. 1, p. 013017, 2011.
